# Managing Persistent Pupillary Membranes With Surgery or Medication: A Report of Three Cases

**DOI:** 10.7759/cureus.86695

**Published:** 2025-06-24

**Authors:** Toshihiko Matsuo, Takehiro Tanaka

**Affiliations:** 1 Division of Healthcare Science, Graduate School of Interdisciplinary Science and Engineering in Health Systems, Okayama University, Okayama, JPN; 2 Department of Ophthalmology, Okayama University Hospital, Okayama, JPN; 3 Department of Pathology, Graduate School of Medicine, Dentistry, and Pharmaceutical Sciences, Okayama University, Okayama, JPN

**Keywords:** anterior segment dysgenesis, cataract, forceps, optical coherence tomography, persistent pupillary membrane, peters anomaly, resection, scissors, vitrectomy cutter

## Abstract

The persistent pupillary membrane, as a congenital anomaly, is a remnant of a network of feeding blood vessels for the lens of the eye, called tunica vasculosa lentis. This study reports three patients with persistent pupillary membrane in both eyes who presented in different situations and were managed differently to achieve better vision. The first child (Case 1) who had been seen initially at the age of two years complained of severe photophobia even though he had good visual acuity, and hence, he and his family chose surgical resection of the pupillary membrane in both eyes at the age of six years just before the admission to an elementary school. He did not develop any surgical complications, such as cataract and glaucoma, and maintained the visual acuity in decimals of 1.2 in both eyes at the age of 17 years.

The second child (Case 2), who was seen first at the age of one month, had persistent pupillary membranes in both eyes, together with Peters' anomaly in the left eye. The iris process adhesion to the corneal inner surface was visualized later by optical coherence tomography. She wore full-correction glasses and obtained the visual acuity of 0.7 in the right eye, so she had no problem studying at an elementary school. She used topical 1% atropine once a week in both eyes to maintain pupillary dilation and also used 0.5% timolol and 1% brinzolamide as pressure-lowering eye drops in the left eye with Peters' anomaly.

The third patient (Case 3) with persistent pupillary membranes in both eyes complained of vision problems for the first time at the age of 49 years when she developed cataract. Surgical resection of the pupillary membrane was done in the initial phase of cataract surgery with intraocular lens implantation in both eyes. At surgical resection of the pupillary membrane, a safe and efficient way was to cut the root of the pupillary membrane on the iris surface with scissors, and then the isolated tissues of the pupillary membrane were pulled out with forceps from the side port at the corneal limbus. Pathological examinations of the excised tissues showed blood vessels with red blood cells in the lumen. In such a rare congenital disease as the persistent pupillary membrane, a case-based approach to choose a better option in different conditions from individual to individual is still required to have a better vision in learning at school and in daily working life.

## Introduction

The persistent pupillary membrane is a rare congenital anomaly and derives from a remnant of a network of feeding blood vessels for the lens of the eye, called tunica vasculosa lentis [1-3]. These embryonic blood vessels that feed the lens tissue regress and disappear until birth. Abnormal status to prevent selective vascular endothelial cells from death with apoptosis, as evidenced by electron microscopic studies, might be an underlying factor for the remnant of the embryonic vascular system [4,5]. The surrounding tissues for the eyeball, called ocular adnexa, are rich with lymphatic vessels, as the other drainage system, which is different from blood vessels [6]. Since the intraocular tissues, such as the lens, iris, ciliary body, retina, and choroid, are lymphatic-free, the persistent pupillary membrane is not designated as related to lymphatic vessels [7].

Due to the rarity of the congenital condition, most of the previous literature references regarding the persistent pupillary membrane are case reports showing the photographs [8-13]. It has been emphasized that an optimal level of visual acuity can be obtained by medical management such as the use of pupil-dilating mydriatic eye drops to widen a pupillary path and the wear of full-correction glasses to avoid ametropic amblyopia [14-17]. In addition, surgical resection of the persistent pupillary membrane, as well as technical aspects of resection has been reported mostly as case reports [18-24]. In this study, we report three consecutive patients with persistent pupillary membranes who have been seen by a single ophthalmologist at a single institution. A case series of these three patients illustrated different clinical situations that were managed differently to obtain a better visual acuity in each individual. We also provided histopathological results of resected pupillary membranes in two patients.

## Case presentation

Case 1

**Figure 1:**
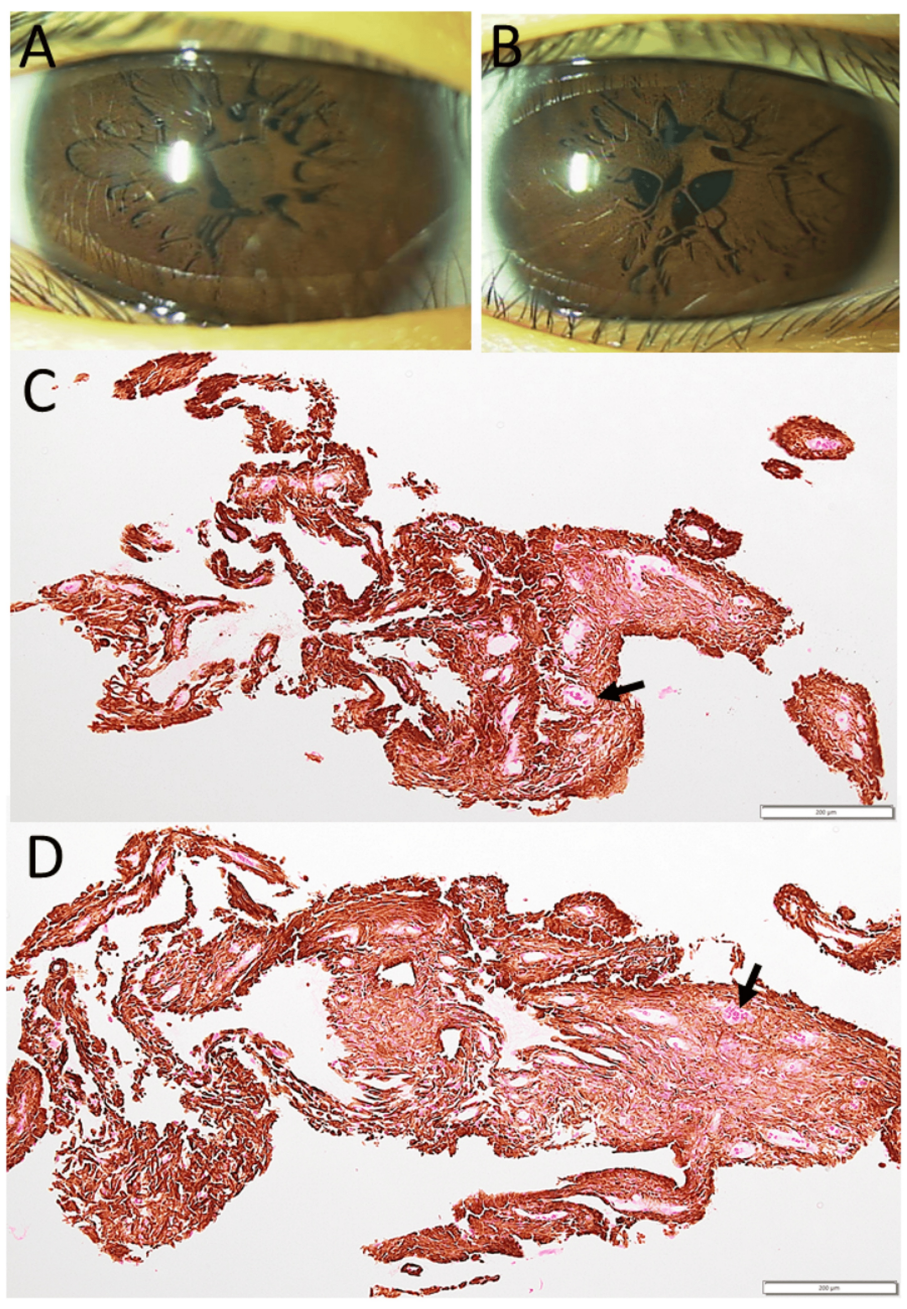
Slit-lamp photographs and pathology of excised pupillary membranes in Case 1 Persistent pupillary membrane in the right eye (A) and left eye (B) at the age of five years. Excised pupillary membrane in the right eye (C) and left eye (D) at the age of six years. Note the vascular lumen with red blood cells (arrows). Hematoxylin-eosin stain. Bar = 200 µm.

A two-year-old boy was brought to an eye doctor with a symptom of photophobia. He had persistent pupillary membranes in both eyes. He was delivered as a breech baby with a birthweight of 3100 g in 39 weeks of gestation with no complications. At the age of three years, the visual acuity in decimals, which was measured separately for the first time in each eye, was 0.4 in the right eye and 0.3 in the left eye. He began to use a topical mydriatic (pupil-dilating) drug (0.5% tropicamide and 0.5% phenylephrine combination) twice daily. At the age of five years, the visual acuity without glasses, both at distance and near, was 0.6 in the right eye and 0.7 in the left eye. He had trouble with severe photophobia in daily life, and his parents wished for surgical intervention (Figures 1A, 1B). At the age of six years, four months before admission to an elementary school, he underwent resection of pupillary membranes in both eyes under general anesthesia (Figures 1C, 1D).

**Figure 2:**
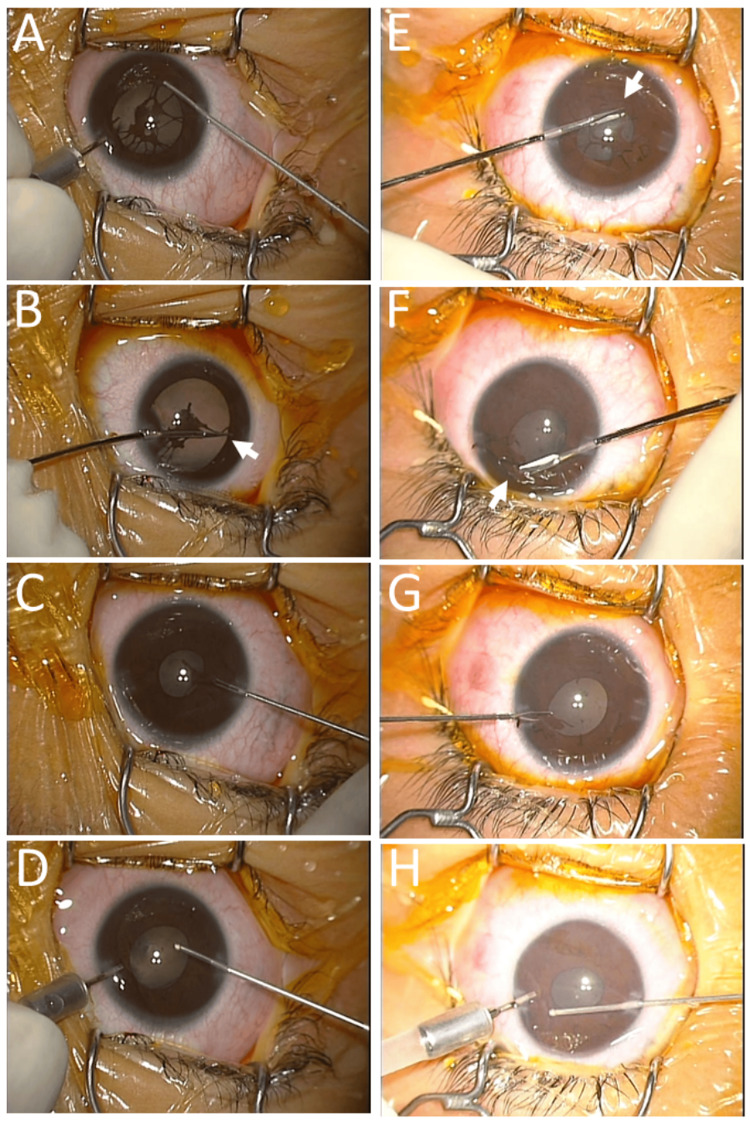
Surgical procedures to excise pupillary membranes (Case 1) Surgical procedures for resection of persistent pupillary membrane at the age of six years. In the right eye, with the time sequence from A, B, C, to D, a 25-gauge vitreous cutter was used under irrigation with a 25-gauge infusion cannula in the first phase (A). Due to technical inefficiency, scissors (arrow, B) were used to cut the roots of the pupillary membrane, and 25-gauge forceps (C) were used to pull out the membrane in the hyaluronate-filled anterior chamber. Finally, the anterior chamber was washed with a vitreous cutter in an aspiration mode (D). In the left eye with the time sequence from E, F, G, to H, scissors were used at the first place to cut the roots on the iris surface (arrows, E, F), the isolated membrane was pulled out with forceps (G), and the anterior chamber was washed finally with a vitreous cutter under the irrigation with an infusion cannula (H).

**Figure 3:**
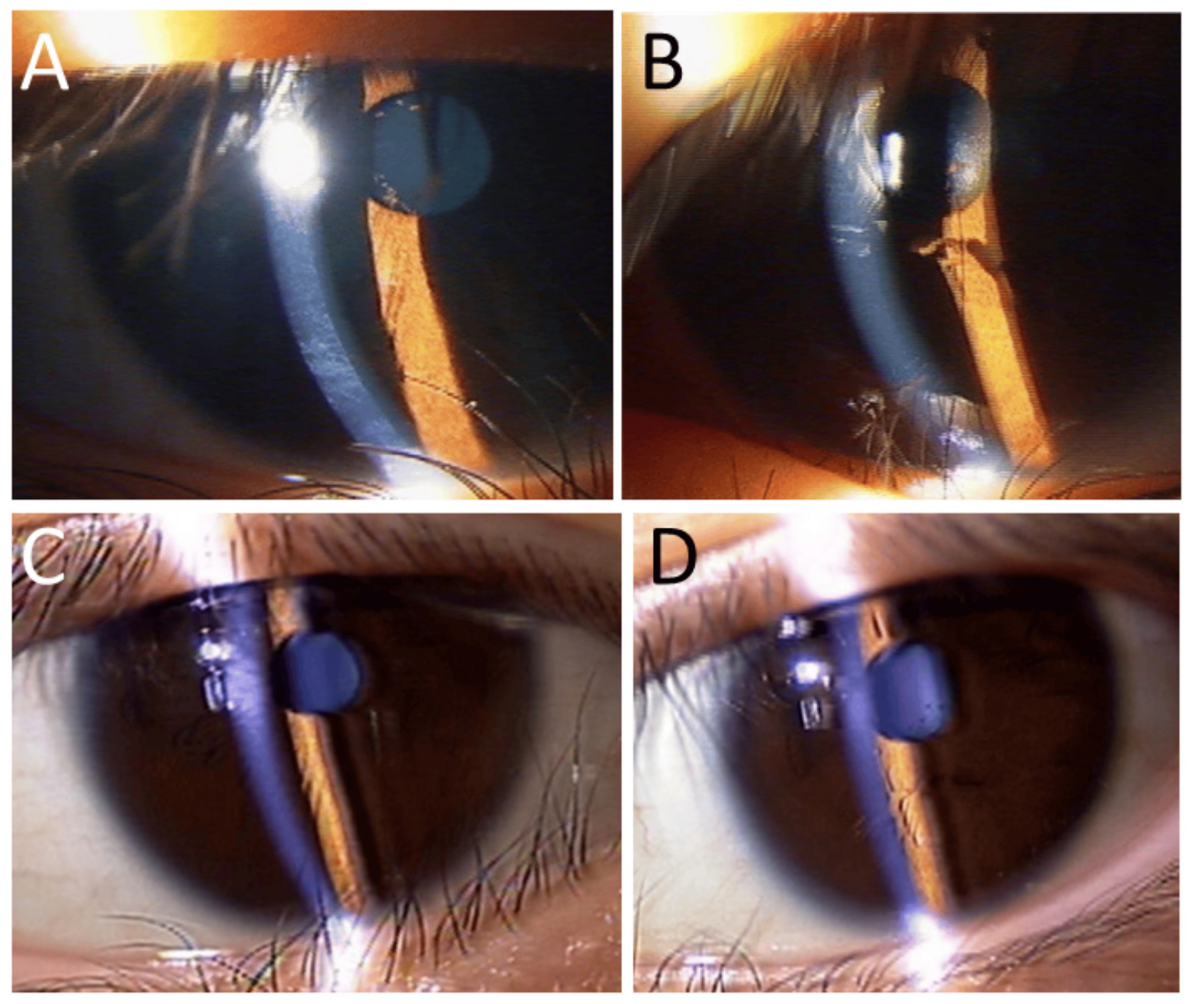
Slit-lamp photographs (Case 1) Slit-lamp photographs in the right eye (A) and left eye (B) on the day after the surgery at the age of six years. Clear cornea and clear lens in the right eye (C) and left eye (D) at the age of 17 years in the last visit.

In the right eye, it took time at the initial approach to excise the pupillary membrane with a 25-gauge vitreous cutter under the irrigation in the anterior chamber with an infusion cannula, both of which were inserted from two side ports at the corneal limbus (Figure 2A). Thus, the anterior chamber was filled with 1% hyaluronate to maintain the space, the roots of the pupillary membrane on the iris surface were cut with scissors (Figure 2B), and the dissected membranes were pulled out with 25-gauge forceps from the side port (Figure 2C). Then, the anterior chamber was washed with a vitreous cutter in an aspiration mode (Figure 2D). Surgical resection in the left eye was done with scissors (Figures 2E, 2F) and forceps (Figure 2G) from the beginning, leading to a shorter surgical time (Figure 2H). He did not develop cataracts or glaucoma (Figures 3A, 3B) and maintained the visual acuity without glasses of 1.2 in both eyes until the last follow-up at the age of 17 years (Figures 3C, 3D).

Case 2

**Figure 4:**
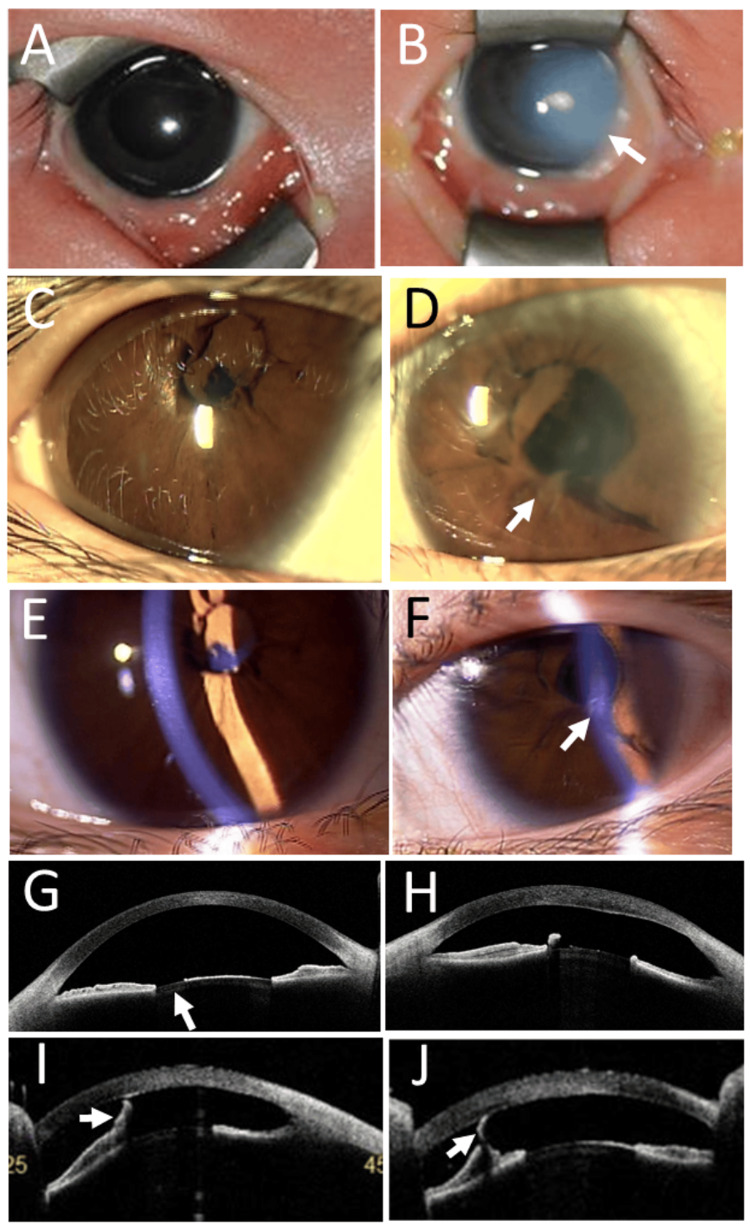
Slit-lamp photographs and optical coherence tomography (Case 2) Photographs at the age of one month at the initial visit, showing persistent pupillary membrane in both eyes (A: right eye, B: left eye), together with a small cornea with temporal-half opacity (arrow) in the left eye (B). Slit-lamp photographs, showing persistent pupillary membranes in both eyes (C: right eye, D: left eye), with topical 1% atropine once a week at the age of nine years. Slit-lamp photographs (E: right eye, F: left eye) and horizontal section images of optical coherence tomography (G: right eye, H: left eye) at the age of 12 years, showing shallow anterior chamber in the left eye (F, H), compared with normal depth in the right eye (E, G). Note the corneal adhesion site (arrows) of the iris process in the left eye (D, F) and open space (arrow) for vision in the pupillary area of the right eye with pupillary membrane (G). Vertical section images (I, J) of optical coherence tomography in the left eye, showing iris process adhesion (arrows) to the corneal inner surface in the lower part. The iris process appears to be linked to the anterior limiting layer of the iris (arrow, I) in a limited resolution of optical coherence tomography.

A one-month-old female baby was brought to an eye doctor because she had persistent pupillary membranes in both eyes. She was delivered with a birthweight of 2668 g in 37 weeks of gestation without any complications in pregnancy. The cornea in the right eye (Figure 4A) was clear in the diameter of 9 mm, while the cornea in the left eye (Figure 4B) showed temporal-half opacity in the smaller diameter of 6 mm. Mydriatic examinations revealed an iris process that extended and inserted into the corneal opacity in the left eye, indicative of Peters' anomaly, in addition to the persistent pupillary membrane. At the age of six months, the cornea in the left eye became clear. She began to have topical 1% atropine, as a pupil-dilating drug, once every week or two weeks, based on the side effects such as salivation. She showed esotropia in the left eye at the age of one year. At the age of three years, she began to wear full-correction glasses, based on cycloplegic refraction with 1% cyclopentolate: Right Eye: 0.3 x cylindrical -2.5 diopters at the axis of 170 degrees, Left Eye: 0.06 x spherical -8.0 diopters and cylindrical -7.0 diopters at the axis of 160 degrees. She began to use 0.5% timolol once daily and 1% brinzolamide twice daily, as intraocular pressure-lowering drugs, in the left eye at the age of 10 years (Figures 4C, 4D). The best-corrected visual acuity with glasses in decimals at near and at distance was 0.7 x cylindrical -1.5 diopters at the axis of 180 degrees in the right eye and 0.06 x spherical -10.0 diopters and cylindrical -2.5 diopters at the axis of 90 degrees in the left eye at the age of 12 years. The intraocular pressure was 16 mmHg in the right eye and 20 mmHg in the left eye. She used topical 1% atropine in both eyes once a week and did not have any vision problems in learning at school. Optical coherence tomography showed the normal depth of the anterior chamber in the right eye (Figures 4E, 4G) and shallow anterior chamber (Figures 4F, 4H) with an iris process adhering to the inner corneal surface (Figures 4I, 4J).

Case 3

**Figure 5:**
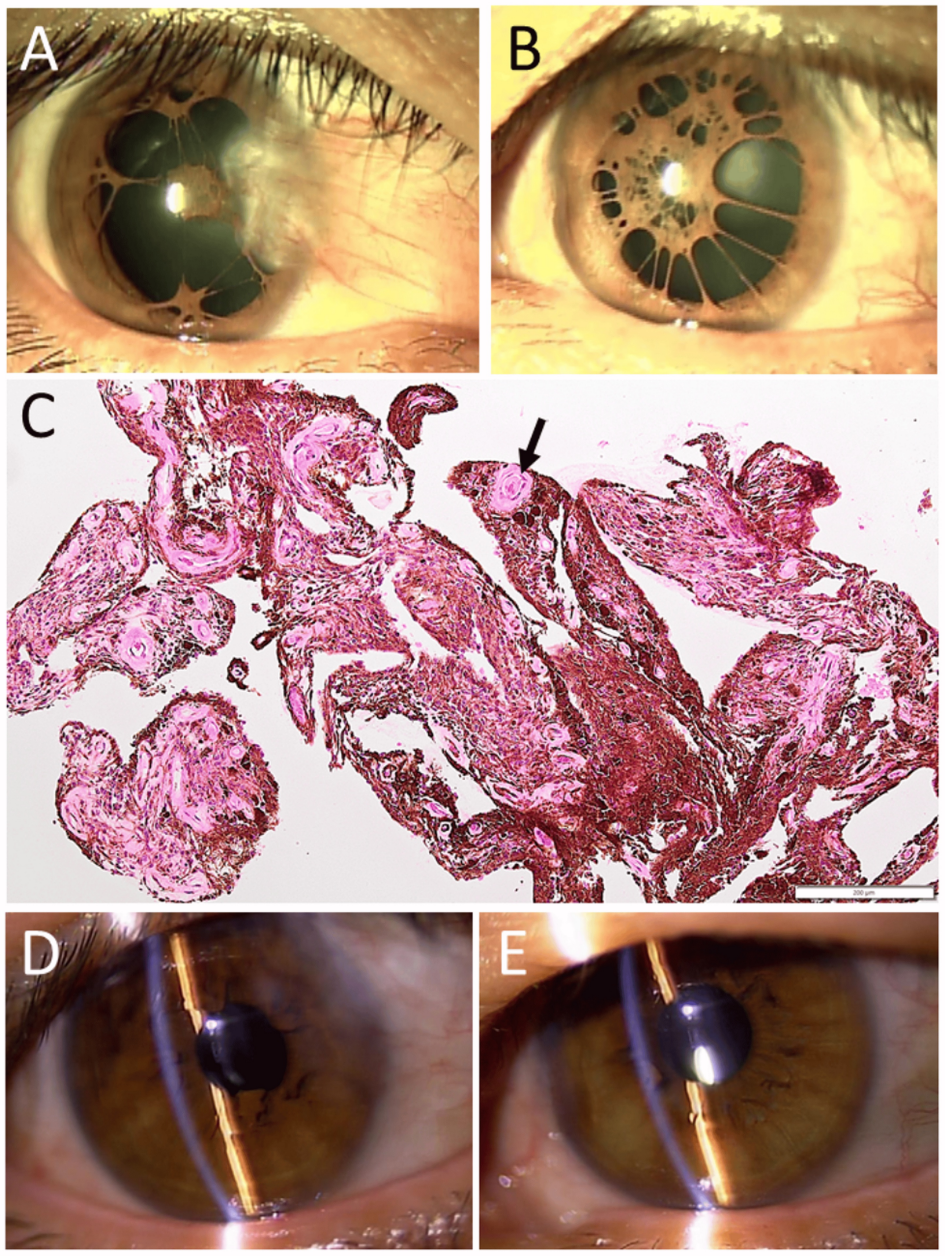
Slit-lamp photographs and pathology of excised pupillary membrane (Case 3) Persistent pupillary membrane with anterior and posterior subcapsular cataract in both eyes (A: right eye, B: left eye) with pterygium in the right eye (A) under mydriasis with 0.5% tropicamide and 0.5% phenylephrine combination eye drops at the age of 49 years. Excised pupillary membrane in the left eye (C) at the age of 56 years. Note the vascular lumen with red blood cells (arrow). Hematoxylin-eosin stain. Bar = 200 µm. Slit-lamp photographs at the age of 57 years, showing clear pupillary areas with intraocular lens implantation in both eyes (D: right eye, E: left eye).

A 49-year-old woman visited an eye doctor with symptoms of decreased vision in both eyes. She had been pointed out to have persistent pupillary membranes in both eyes in childhood. She took escitalopram 5 mg daily for anxiety, olmesartan 5 mg and amlodipine 5 mg daily for hypertension. She did not have other past history. The best-corrected visual acuity in decimals was 0.1 in the right eye and 0.2 in the left eye. She had anterior and posterior subcapsular cataracts in both eyes (Figures 5A, 5B), which were designated as a cause for recent vision problems. The right eye had pterygium (Figure 5A). The fundus examinations with mydriasis were normal in both eyes. She did not wish to have surgery due to anxiety, and thus, glasses for myopia were prescribed. At the age of 56 years, she decided to undergo cataract surgery with intraocular lens implantation separately in the sequence of the right eye and left eye, with an interval of one week. The pupillary membrane was cut with scissors and pulled out with forceps in the hyaluronate-filled anterior chamber in the first phase of surgery (Figure 5C). Cataract surgery was then performed as usual. The best-corrected visual acuity with myopic corrections was 0.5 in both eyes at the last follow-up, one year after the surgery (Figures 5D, 5E).

## Discussion

Table 1 summarizes the three patients presented in this study. Case 1 patient might not have to undergo the resection of persistent pupillary membranes in both eyes since he had the visual acuity of 0.6 or better in both near viewing and distant viewing. A severe symptom of photophobia in daily life made him and his family decide to have surgical resection before admission to an elementary school. Case 2 patient showed a small cornea with an iris process adhering to the corneal inner surface in the left eye, indicative of Peters' anomaly, in the presence of persistent pupillary membranes in both eyes. The situation in the left eye resulted naturally in poor visual acuity and was not indicated for surgical intervention. She had the visual acuity of 0.7 in the right eye with no problem in learning, and thus, was observed with topical 1% atropine once a week. Case 3 patient had a decrease in the visual acuity in both eyes because of cataract progression and underwent cataract surgeries, as well as pupillary membrane resection in both eyes to gain the visual acuity of 0.5 in both eyes.

**Table 1:** Summary of three patients with persistent pupillary membrane in both eyes D, diopters; cyl., cylindrical.

Case/Sex/Age at first visit	Eye	Visual acuity at first measurement and age	Clinical features	Visual acuity at last measurement and age
1/Male/Two years	Right	0.4 with no correction, at age three years	Severe photophobia, Pupillary membrane resection at age 6 years	1.2 without correction, at age 17 years
	Left	0.3 with no correction, at age three years	Severe photophobia, Pupillary membrane resection at age 6 years	1.2 without correction, at age 17 years
2/Female/One month	Right	0.3 with cyl. -2.5D at axis 170, at age three years	No surgery, Topical 1% atropine once a week at age 6 months	0.7 with cyl. -1.5D at axis 180, at age 12 years
	Left	0.06 with -8.0D and cyl. -7.0D at axis 160, at age three years	Small cornea with Peters' anomaly, No surgery, Topical 1% atropine once a week at age 6 months, Topical 0.5% timolol and 1% brinzolamide at age 10 years	0.06 with -10.0D and cyl. -2.5D at axis 90, at age 12 years
3/Female/49 years	Right	0.1 with -3.0D and cyl. -2.0D at axis 180, at age 49 years	Cataract surgery with pupillary membrane resection at age 56 years	0.5 with -2.5D and cyl. -1.5D at axis 180, at age 57 years
	Left	0.2 with -6.5D and cyl. -2.0D at axis 180, at age 49 years	Cataract surgery with pupillary membrane resection at age 56 years	0.5 with -4.0D and cyl. -2.5D at axis 180, at age 57 years

The pupillary membranes have to be excised in the first place to proceed to cataract surgery. In the situation of cataract surgery as shown in Case 3, the persistent pupillary membranes can be resected safely with scissors in the hyaluronate-filled anterior chamber, with no concern for damage to the anterior surface of the lens capsule. In contrast, as in Case 1, every care is taken not to touch the anterior surface of the lens capsule with scissors when the pupillary membrane is cut in a child who has a clear lens with a normal level of accommodative power. Based on his experience in congenital cataract surgery [25], the author Toshihiko Matsuo, as a surgeon, thought that surgical removal of the pupillary membrane with a 25-gauge vitreous cutter under the irrigation with a 25-gauge infusion cannula in the anterior chamber would be more efficient than cutting with scissors. However, that was not the case. Therefore, he changed the surgical strategy to cut the pupillary membrane with scissors with the aid of hyaluronate in the anterior chamber. As reported in a case series by Lim and Yu, no cataract was observed in this child (Case 1) until adulthood [19].

Topical use of a pupil-dilating (mydriatic) drug to widen the pupillary area for the optical path is one option for the medical treatment of persistent pupillary membrane. Case 1 patient complained of photophobia before the topical use of 0.5% tropicamide and 0.5% phenylephrine combination twice daily, and he continued to have photophobia after the age of three years when he started to use the topical mydriatics. He did not complain of photophobia after the resection of pupillary membranes in both eyes, indicating that photophobia was due to the pupillary membranes by themselves. He also showed a visual acuity of 1.2 in both eyes after the surgery, indicating no presence of amblyopia. Visual field assessment, photophobia score, or disability scale, although not done in this boy, might help provide more scientific evidence for surgical indication.

Case 2 girl began to use topical 1% atropine once a week at the age of six months and continued to use it until the latest visit at the age of 12 years. She did not complain of photophobia throughout the course. Case 3 patient did not notice any vision-related symptoms until adulthood, when she developed cataracts. As shown in Case 1 and Case 2, a topical pupil-dilating drug can be used as a treatment option to avoid the development of amblyopia, together with wearing full-correction glasses [14-17]. It should be emphasized that a risk-benefit assessment is mandatory to proceed to the resection of the persistent pupillary membrane since the incidence of long-term complications such as cataract and glaucoma remains unknown.

Optical coherence tomography to visualize sectional images for anterior segments of the eye has become available at ophthalmic practice as a short-time and non-invasive examination to show the depth of the anterior chamber, iridocorneal angle width, corneal and iris structural abnormalities, including persistent pupillary membrane [26, 27]. In Case 2, iris process adhesion to the corneal inner surface was visualized by optical coherence tomography, which supports the diagnosis of Peters' anomaly in the presence of persistent pupillary membrane. Peters' anomaly is in the spectrum of congenital diseases called anterior segment dysgenesis, namely, a group of congenital eye abnormalities that affect the front part of the eye, such as the cornea and iris, and has been reported to be associated with the persistent pupillary membrane [28,29]. Although persistent pupillary membranes and Peters' anomaly may coexist, they represent distinct embryological entities. Peters' anomaly is a form of anterior segment dysgenesis characterized by central corneal opacity, iridocorneal adhesions, and defects in Descemet’s membrane and the posterior stroma [28,29]. In contrast, the persistent pupillary membrane results from incomplete regression of the tunica vasculosa lentis [1,2,4]. In Case 2, the presence of an iris process adherent to the posterior cornea and a smaller corneal diameter would support the diagnosis of Peters' anomaly rather than an atypical persistent pupillary membrane extension.

A coexisting malformation of the trabecular meshwork, as an aqueous outflow pathway, predisposes patients with anterior segment dysgenesis to develop glaucoma at a higher rate. The intraocular pressure in both eyes remained in the normal range throughout the course in Case 2, with the aid of pressure-lowering eye drops in the left eye with Peters' anomaly. The anterior-segment optical coherence tomography, which we used in this study, could not visualize a more precise structure of the persistent pupillary membranes and Peters' anomaly in Case 2.

Optical coherence tomography with a high resolution may be useful to visualize the relation of the pupillary membrane with the iris [30]. While our anterior-segment optical coherence tomography images provided useful anatomical relationships, we did not apply pseudocolor-rendering techniques. Recent studies have shown that pseudocolor anterior-segment optical coherence tomography enhances the detection of iris layer abnormalities, particularly the anterior limiting layer signal attenuation in disease states [30]. Future imaging with higher-resolution, intensity-mapped anterior-segment optical coherence tomography could offer better structural correlation with histology.

**Figure 6:**
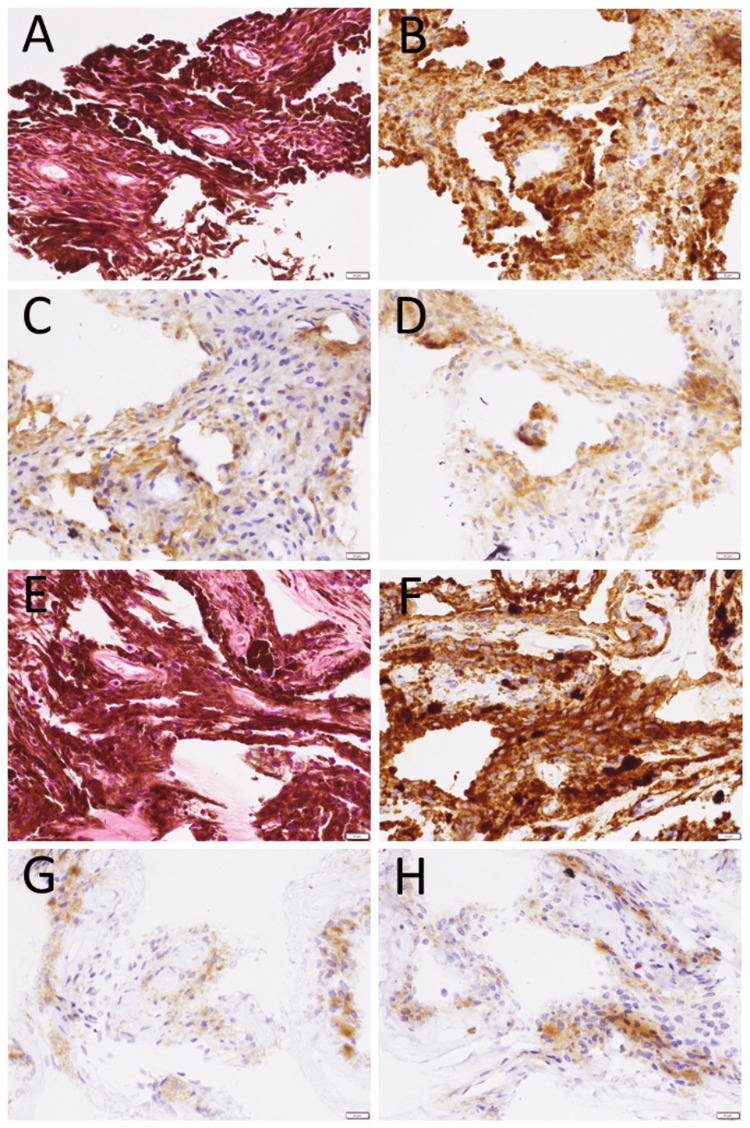
Immunostaining of resected pupillary membranes Immunostaining of the resected pupillary membrane in the left eye of Case 1 (A-D) and the left eye of Case 3 (E-H) with hematoxylin-eosin stain (A, E). Immunostaining with diaminobenzidine (DAB) after melanin breach with 20% hydrogen peroxide, showing melanin-baring cells positive for D2-40 (B, F), marker for lymphatic vessels, in contrast with vascular lumen-lining cells negative for D2-40 (B, F), positive for CD31 (C, G) and ERG (D, H), markers for vascular endothelial cells. CD31-positive and ERG-positive cells are arranged in a fascicular pattern to show probably a longitudinal section of vessels, although the staining would be an artifact due to the remaining melanin. Bar = 20 µm.

The pathological examinations of resected pupillary membranes in Case 1 and Case 3 showed vascular structures with red blood cells in the lumen, which were comprised in the gathering of pigment-bearing cells in hematoxylin-eosin stain. The presence of vascular structures supports the fact that the persistent pupillary membrane is the remnant of fetal vasculature, which feeds the formation of the lens in the eye [1,2]. It should be noted that the vascular lumen in the persistent pupillary membrane had red blood cells, which could not be seen clinically. To search further for the vascular nature of persistent pupillary membranes, immunostaining was done after melanin breach with 20% hydrogen peroxide in the preserved specimens of Case 1 and Case 3, and revealed that vascular lumen-lining cells were positive for CD31 and ERG (Ets-related gene), both of which are markers for vascular endothelial cells, while were negative for D2-40 (podoplanin), a marker for lymphatic vessels. Most pigment-bearing cells in the resected pupillary membranes after the melanin breach were positive for D2-40, in contrast with the vascular lumen-lining cells negative for D2-40 (Figure 6). CD31-positive and ERG-positive cells were arranged in a fascicular pattern, which probably represents a longitudinal section of vessels, although the staining would be an artifact due to the remaining melanin. In a previous study, lymphatic vessels stained with D2-40 appeared to be absent in the intraocular tissue of the human fetal eyes, in contrast with the conjunctival surface, which had lymphatic vessels [31]. In other previous studies, podoplanin (D2-40)-positive cells have been reported to be present at the anterior border of the iris of the human eye [32,33]. The immunostaining in this study supports the notion that the persistent pupillary membrane is related to the anterior border of the iris.

Embryologically, the iris stroma and anterior limiting layer arise from neural crest-derived mesenchyme, whereas the pupillary membrane originates from mesodermal vascular elements [1,2,28,29]. Although the two are derived from different germ layers, their anatomical proximity during fetal development could explain structural associations observed in persistent membranes. The presence of podoplanin-positive pigment-bearing cells near the pupillary remnants supports a relationship with the anterior iris border, but further work is required to map this precisely.

## Conclusions

The persistent pupillary membrane is a rare congenital anomaly, and the management may be changed in each individual to obtain better vision. The first child (Case 1) complained of severe photophobia even though he had good visual acuity, and hence, he and his family chose surgical resection of the pupillary membrane in both eyes at the age of six years before admission to an elementary school. He did not develop any surgical complications such as cataract and glaucoma until the age of 17 years. The second child (Case 2) had persistent pupillary membrane in both eyes, together with Peters' anomaly in the left eye, which was visualized later by optical coherence tomography. She showed good visual acuity in the right eye with no problem in studying at an elementary school. The third patient (Case 3) with persistent pupillary membrane in both eyes complained of a decrease in visual acuity for the first time at the age of 49 years when she developed cataract. Surgical resection of the pupillary membrane in both eyes was done at the beginning of cataract surgery with intraocular lens implantation. At the surgical resection of the pupillary membrane, a safe and efficient way was to cut the root of the pupillary membrane on the iris surface with scissors, and then the isolated tissues of the pupillary membrane were pulled out with forceps from the side port at the corneal limbus.

A major limitation in this study is the small sample size and thus, the lack of generalizability due to the rarity of persistent pupillary membrane. Additionally, functional vision assessment tests were not performed in the present series of patients in this study. From a broader perspective, different clinical pictures of each patient will hint to clinicians who encounter such a rare congenital anomaly as persistent pupillary membrane. As a matter of course, these cases underscore the individualized approach required in managing persistent pupillary membranes. Correlative analysis of anterior-segment optical coherence tomography with histological features, particularly regarding the anterior limiting layer, may enhance our understanding of these anomalies.
